# Combined Effect of Synthetic and Natural Polymers in Preparation of Cetirizine Hydrochloride Oral Disintegrating Tablets: Optimization by Central Composite Design

**DOI:** 10.1155/2017/8305976

**Published:** 2017-01-05

**Authors:** Chandra Sekhar Patro, Prafulla Kumar Sahu

**Affiliations:** Raghu College of Pharmacy, Dakamarri, Visakhapatnam, Andhra Pradesh 531 162, India

## Abstract

Our aim was to employ experimental design to formulate and optimize cetirizine hydrochloride oral disintegrating tablets (ODTs) by direct compression technique, using the mutual effect of synthetic croscarmellose sodium (CCS) and natural* Hibiscus rosa-sinensis* mucilage (HRM) as disintegrants in the formulation. Central composite design (CCD) was applied to optimize the influence of three levels each of CCS (*X*_1_) and HRM (*X*_2_) concentrations (independent variables) for investigated responses: disintegration time (DT) (*Y*_1_), % friability (*F*) (*Y*_2_), and % cumulative drug release (DR) (*Y*_3_) (dependent variables). This face-centered second-order model's reliability was verified by the probability and adequate precision values from the analysis of variance, while the significant factor effects influencing the studied responses were identified using multiple linear regression analysis. Perturbation and response surface plots were interpreted to evaluate the responses' sensitivity towards the variables. During optimization, the concentrations of the processed factors were evaluated, and the resulting values were in good agreement with predicted estimates endorsing the validity. Spectral study by Fourier Transform Infrared Spectroscopy (FTIR) and thermograms from Differential Scanning Calorimetry (DSC) demonstrated the drug-excipients compatibility of the optimized formulation. The optimized formulation has concentrations of 9.05 mg and 16.04 mg of CCS and HRM each, respectively, and the model predicted DT of 13.271 sec, *F* of 0.498, and DR of 99.768%.

## 1. Introduction

The conventional formulations like tablets and capsules play a major role in the oral drug-delivery system with many pros and cons. With respect to patient compliance, ease of swallow is one of the important factors that determine the acceptance of these formulations, especially in pediatric and geriatric patients. It was assessed that 40–50% of the population face the problem of dysphagia or difficulty in swallowing with frequent complaints of taste, surface, and size of the tablets which lead to noncompliance and poor treatment [1–3]. Technological advents in ODTs have drawn global attention during the last decades that can overcome these problems. Unlike conventional dosage forms, ODTs rapidly disintegrate in the mouth in presence of saliva and are then swallowed comfortably into the stomach [4–7]. The drug release from ODTs has a prospect to be absorbed in oromucosal tissue followed by esophagus and pharynx resulting in potential rapid action, enhanced therapeutic efficacy devoid of gastric irritation, and partial first-pass effect [8].

For an ideal ODT, the disintegration time varies from several seconds to about a minute. Numerous unique properties of ODTs like fast disintegration, taste-masking ingredients, sensitiveness to moisture, tablet strength, and porosity make them distinct from conventional tablets. However, the best formula of ingredients' composition to achieve the desired properties has been a great challenge for the researchers since time immemorial. Recently, Response Surface Methodology (RSM) has become a widely accepted optimization technique to overwhelm the complexity in formulation and development of pharmaceutical preparations [9]. The aim of RSM is to design the way in which a response is affected by the independent variables or their interactions. The fitted model is used to reach the destination at the best operating conditions, which conclude in either maximum or minimum response. It is also useful to analyze the functional relation between completely dependent and wholly autonomous variables [10]. RSM has many types of experimental designs, which demonstrates polynomial equations and determines the optimal levels to formulate the dosage forms [11–13].

CTZ, an active hydroxyzine (H1-receptor antagonist) metabolite, is a drug of choice for the treatment of all types of allergies, rhinitis, hay fever, atopic dermatitis, asthma, allergic cough, and urticaria. Being a second-generation nonsedative antihistamine, it exhibits inhibition of several cytotoxic mediators, eosinophil chemotaxis, and release of histamines during allergies [14, 15]. In this paper, we have demonstrated the formulation and development of cetirizine hydrochloride (CTZ) ODTs using an optimized combination of CCS and HRM as disintegrants. Disintegration capacity of the natural mucilage (HRM) was studied when employed alone and in binary mixture with the synthetic superdisintegrant (CCS) and vice versa. During comparative evaluation, it was observed that the ODTs with optimal combination of CCS and HRM provide faster disintegration and better tablet strength.

## 2. Materials and Methods

### 2.1. Materials

CTZ was procured from Lotus Enterprises, Visakhapatnam (India). CCS, Pearlitol SD 200, magnesium stearate, sorbitol, aerosil, and flavour were purchased from Yarrow Chem Products, Mumbai, and aspartame was purchased from Loba Chemicals, Mumbai. Fresh leaves of* Hibiscus rosa-sinensis* were collected from the local source.

### 2.2. Methods

#### 2.2.1. Extraction and Purification of* Hibiscus rosa-sinensis* Mucilage

The fresh and healthy leaves of* Hibiscus rosa-sinensis* were carefully cleaned. The dirt and dust particles were removed by washing with water, dried, and processed. Powdered leaves were set aside in water for a period of 5-6 hrs for soaking followed by boiling for 30 min. The mucilage was collected into water from the above mixture. Thereafter, the material was squeezed to remove the marc from the solution by an eight-folded muslin cloth bag. A sufficient quantity of acetone was added to the above filtrate to get the precipitate. The collected mucilage was dried at a temperature of 30°C in hot air oven. The dried mucilage was powdered, sieved through sieve (#80), and put aside in a desiccator at 30°C and 45% relative humidity until use. The common method of segregation of gums from food was used. About 1% of mucilage along with 5% of cold diluted trichloroacetic acid solution was homogenized, centrifuged, and neutralized by using sodium hydroxide and then dialyzed for 30 hrs against distilled water. The mucilage was reprecipitated using ethanol (three volumes) and washed successively with ethanol, acetone, and diethyl ether [16, 17]. The dried powder mucilage was characterized for physicochemical properties shown in Table 1.

#### 2.2.2. Drug: Excipient Compatibility Study

Both FTIR and DSC analysis was carried out to evaluate the interfering between drug and excipients used for the CTZ ODTs formulation.

#### 2.2.3. Formulation Development

CCS, a synthetic polymer, and natural mucilage of HRM were individually used as disintegrating promoting agents for preparing oral disintegrating tablets. Four different concentrations of the respected disintegrants were discreetly chosen for the formulation development. Each preliminary trial batch of the formulation was composed of various proportions of drug and excipients as depicted in Table 2. ODTs of each batch of 50 tablets (each tablet weight is 200 mg ± 50 mg) were prepared by direct compression method using Cadmach single punch machine with 10 mm flat plane face punches. The drug and excipients sieved through #22 mesh and mixed accurately in a polyethylene bag for 30 minutes. To the resultant blend, lubricant was added and mixed well to get the uniform composite. The formulations were equipped to develop the tablets. Although additions of natural mucilage have shown satisfactory results, the formulations containing synthetic polymer exhibited better performance when evaluated [18, 19].

Further, a combination of both the above disintegrants in different ratios was screened to assess their mutual contribution on the formulation's performance. The proposed method aimed to establish a formulation containing an optimal ratio of both the disintegrants to exhibit their best synergistic effects on the investigated responses.

#### 2.2.4. Formulation Design

To develop and optimize the formulation design of CTZ ODTs, a CCD with *α* = 1 was used to recognize the significant factors' effects influencing the investigated responses in the proposed oral disintegrating tablet formulation. The concentrations of CCS (*X*_1_) and HRM (*X*_2_) as independent variables run at three levels were discreetly screened for their major effect and interactions on the responses such as DT (*Y*_1_), *F* (*Y*_2_), and DR (*Y*_3_) as dependent variables. The responses were optimized together by multiple response algorithms using Design-Expert® version-8.0.4 (Stat-Ease). To depict the interrelationship between independent and dependent variables, the investigational data and model were fitted and evaluated by ANOVA.

As per the above-mentioned factors, a CCD was used, where the two independent factors converted to being dimensionless each at three levels (+1, 0, −1) to have control over the response pattern and their optimum variable combinations. The central point (0, 0) of the design was studied in quintuplicate to compute the reproducibility of the technique and also to countenance the valuation of error.  Table 3 summarizes a version of 13 experimental runs, their independent variable's combination, and coded level version used during study.

During the design study, all the responses develop fitted polynomial models, along with their interactions and quadratic expressions utilizing multiple regression analysis methodologies. The fitting form of the second-order polynomial model is described as the following equation:

(1) Y=β0+β1X1+β2X2+β3X1X2+β4X12+β5X22+β6X1X22+β7X12X2

*Y* is the predicted/measured response for the combination of each factor level, which correlates with *β* (regression coefficient): *β*_0_, which is the intercept signifying the arithmetical mean of whole of quantifiable results of 13 runs; *β*_1_ to *β*_7_ are linear coefficients appraised from the contemplated values of the measured response; *X*_1_ and *X*_2_ are translated coded values for each independent variable. The expressions *X*_1_ *X*_2_ and *X*_*i*_^2^ signify the interaction between them and influence on response. The rationality of statistical polynomial models was predictable by ANOVA. Three-dimensional response surface plots (3D) were designed to check the interaction of factors and their significant influence on responses [20–22].

#### 2.2.5. Validation and Optimization of Proposed Model

To validate the experimental design, eight checkpoint solutions were selected for investigation. The prepared formulations equivalent to each of the checkpoints were screened for the selected responses. The resultant observed responses were compared quantitatively with their corresponding foretold values. Subsequently, the linear regression plots were drawn between the obtained observed response properties and the consequent predicted values to observe the error.

## 3. Evaluation

### 3.1. Mechanical Properties of Tablets

To determine the mechanical strength of a tablet, hardness and friability are measured as the two significant parameters. The crushing strength/hardness of the tablets was measured by using the hardness tester (Monsanto), whereas the friability *F* was evaluated using a Roche friabilator. For % friability, accurately weighed twenty tablets were allowed to rotate in the friabilator at 25 rpm for 5 minutes and change in tablet weight was analyzed.

### 3.2. Wetting Profile

The wetting time of the formulations was calculated by standard procedure. Five circular pieces of filter papers of 10 cm diameter and 0.45 *μ*m pore sizes (Hi-media) were placed in a Petri dish. 10 mL of eosin dye water solution was added to the dish; then a tablet was positioned on the filter paper and time taken for overall wetting of the tablet was noted down [23].

### 3.3. Disintegration Test of Tablets

The USP disintegration apparatus is having six glass tubes 3′′ long with top side open and detained beside 10′′ screen at the opposite bottom side of the basket. After the tablet is sited in every tube, the basket frame is disillusioned in one-liter beaker of double distilled water at 37 ± 2°C, in such a manner that the tablets stay behind the liquid surface on their uphill movement and downward not nearer than 2.5 cm from the bottom of the basket. The DT was recorded [24].

### 3.4. In vitro Dissolution Study

The ODTs were evaluated for drug release studies by using phosphate buffer (pH-7.4) for one hour to contact the capability of the formulated tablets to furnish quick drug delivery. The eight-stage dissolution test apparatus (DISSO 2000, Lab India) was used to perform in vitro drug dissolution studies of ODTs by using 900 mL of the dissolution medium (pH-7.4 phosphate buffer) constantly well kept at 37±1°C. The tablets were placed in the cylindrical vessel, and the paddle type stirrer rotated at 50 rpm. Samples of 5 mL were withdrawn at each time interval (2, 5, 10, 15, 20, 30, 45, and 60 minutes) from the dissolution medium and replaced by 5 mL of fresh mediums each time to maintain sink condition. The drawn samples were filtered, and 1 mL was taken of each filtrate to dilute to 10 mL with same media. The absorbance of the samples was measured at *λ*_max_ 250 nm using UV spectrophotometer [25].

## 4. Results and Discussion

### 4.1. Drug-Excipients Interaction Study

**Figure 1:**
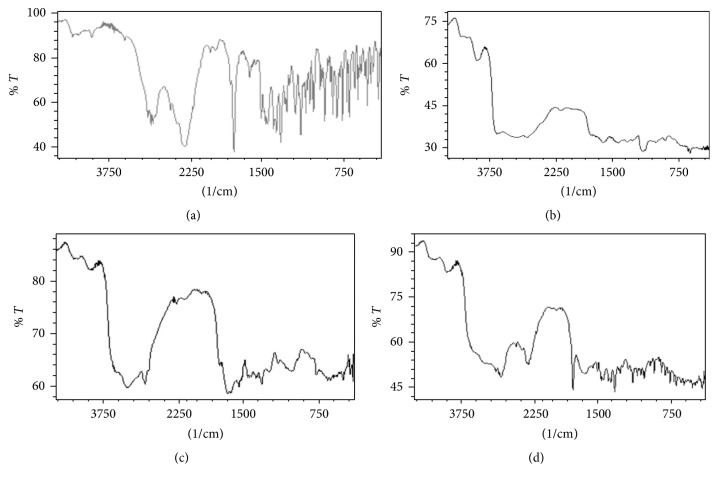
FTIR spectra for (a) cetirizine; (b) physical mixture of cetirizine with CCS; (c) physical mixture of cetirizine with HRM; and (d) optimized formulation, physical mixture of cetirizine with CCS and HRM.

The interaction study between drug and excipients was carried out in order to get confirmation on the probable interaction for any interface using FTIR and DSC analysis.  Figure 1 shows the infrared spectra of pure CTZ, CCS, and HRM and combination of these three ingredients. The unadulterated drug alone shows 3043, 3022, 2983 (aromatic C-H str), 1600, 1580 (aromatic (C⋯C)), 1741 (C=O), 1200–1100 (C-O-C), 1250–1310 (C-N), 2891–2741 (C-OH) (w, b), and 750–700 (C-Cl), respectively. The optimal ratio of CCS and HRM as the key excipients in the formulation showed almost all bands without affecting their peak position and trends, which indicates the absence of well-defined interaction between the drug and the two disintegrating agents.

**Figure 2:**
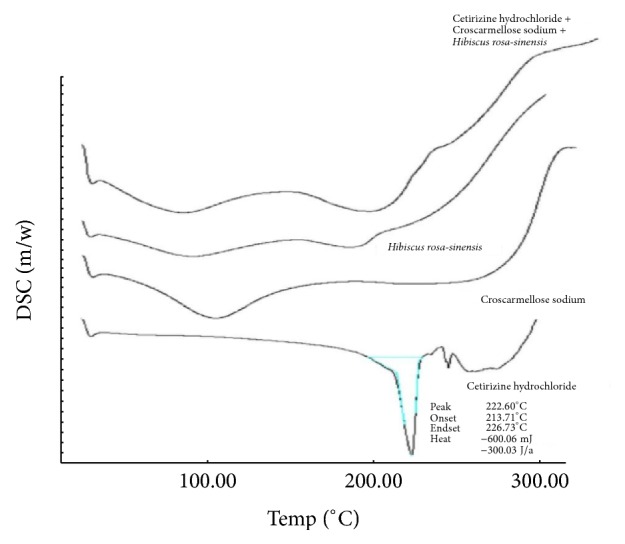
Overlaid DSC curve of cetirizine HCl, CCS, HRM, and their mixture.

The DSC thermograms of pure CTZ, CCS, and HRM and combination of these three are shown in Figure 2. The thermogram of pure CTZ exhibits a single endotherm corresponding to the melting point of the pure drug, which showed a sharp characteristic peak at 222.6°C (Tonset = 213.71°C, Tendset = 226.73°C, and heat of fusion is 600.06°C) due to melting point of the solid drug. The thermogram of mixture did not show any peak at 222.6°C, which confirms compatibility of drug and excipients. There is no melting endotherm of drug in the optimized formulation, which indicates that the drug was present in amorphous phase.

### 4.2. Experimental Design and Data Acquiring

The influence of both disintegrants used to formulate CTZ ODTs was studied. Tablets were obtained employing direct compression method due to (i) ease of fabrication and (ii) economic (iii) faster disintegration or dissolution. HRM was used as disintegrating promoting agent due to its swelling property in water. After coming in contact with water the mucilage wicks the water into the matrix network and then swells, which reduces adhesiveness of other ingredients causing disintegration. CCS used as disintegrating agent due to its cross network polymeric system water drawing capacity considerably increases. RSM with the aid of CCD was exploited methodically to estimate the impact of disintegrants as dependent variables and their interactions on the investigated responses. The experiment was aimed to identify the significant factor effects influencing the formulation performance and to establish their superlative levels for the desirability of responses shown in Table 4.

### 4.3. Statistical Analysis and Mathematical Modeling of Experimental Data

To estimate the quantitative effects of the combined ratio of factors and their levels on the selected responses, the experimental values of the flux were analyzed by Design-Expert software and mathematical models obtained for each response [26]. The statistical models were generated by the results obtained from investigation and regression of statistically significant factors [8]. The polynomial equations derived from multiple regression analysis for each flux are shown below.

(2) Y1Disintegration time=+12.38−0.50∗X1−0.50∗X2+2.50∗X1∗X2+1.67∗X12+4.67∗X22−1.00∗X12∗X2+0.00∗X1∗X22

(3) Y2% Friabilty=+0.48−0.025∗X1−0.050∗X2+0.057∗X1∗X2+0.046∗X12−0.089∗X22−0.022∗X12∗X2−0.062∗X1∗X22

(4) Y3Cumulative % drug released=+100.33+0.57∗X1+0.32∗X2−0.75∗X1∗X2−0.12∗X12−1.75∗X22−0.047∗X12∗X2−0.40∗X1∗X22

The above equations reveal the assessable effect of the dependent variables, concentrations of CCS (*X*_1_) and HRM (*X*_2_) and their interaction on the responses such as DT (*Y*_1_), *F* (*Y*_2_), and DR (*Y*_3_) as dependent variables. The polynomial equation includes the coefficients intercept, first order of individual factor's influence, interaction, and higher-order term [27]. In the equations, the positive signs indicate synergistic effect, and the negative sign signifies the antagonistic affect. The negative regression coefficient of both factors (*X*_1_ and *X*_2_) in (2) and (3) proposes a decrease in DT and *F* with an increase in concentration of the independent variables and in (4) an increase in DR with an increase in concentrations of factors. It is also concluded that variable *X*_2_ had the most profound effect on % friability, whilst variable *X*_1_ mostly affected % drug release. However, disintegration time experienced equal influence by both the variables (*X*_1_ and *X*_2_). In (2)–(4) coefficients of factors with higher-order term (*X*_1_^2^, *X*_1_^2^) represent quadratic correlation, while the coefficients having both factors (*X*_1_, *X*_2_) indicate an interaction effect on the selected responses. The positive regression coefficient of the quadratic term of *X*_1_^2^ in (2) and (3) signifies that the respective responses decrease slightly and later increase, whereas in (4) negative indicates the decrease in drug release. The quadratic term *X*_2_^2^ had significant effect on all the three responses (disintegration time, % friability, and % cumulative drug release). There is a positive influence on both DT and *F* by the interaction of both factors and negative impact on drug release [28, 29].

The analysis of variance (ANOVA) test on the quadratic response model was executed to signify the linear interaction effect of the factors, the quadratic term on responses, and lack of fit [30]. The analysis of variance (ANOVA) was calculated by the Design-Expert software as shown in Table 5. At the significance level of 5%, the quadratic model was significant, as the *p* value is less than 0.05 [31].

### 4.4. Evaluation of Variable Effectiveness by Perturbation Plots

**Figure 3:**
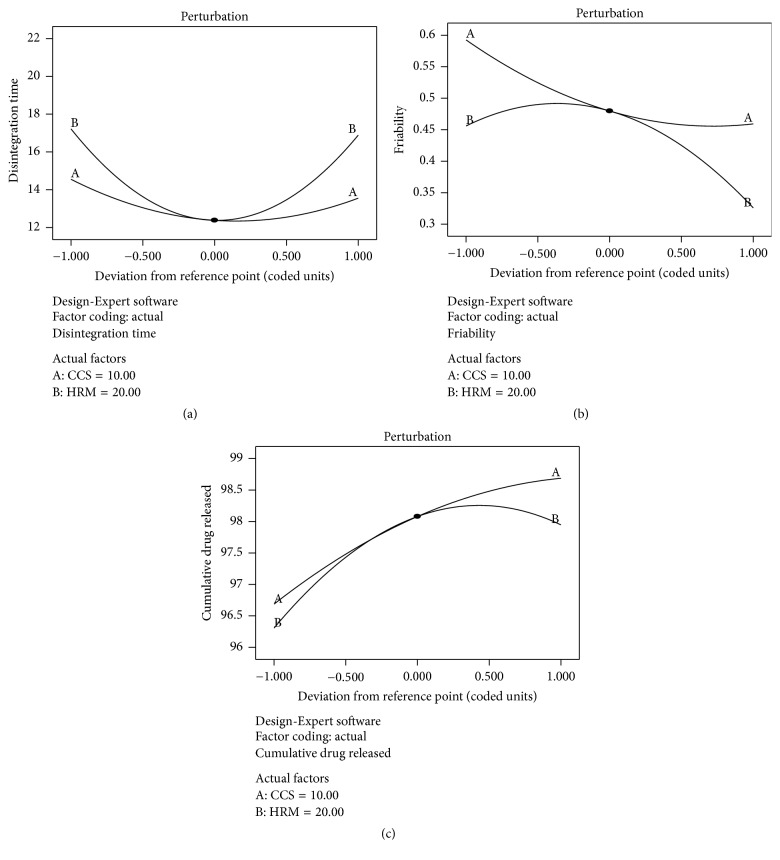
Perturbation plots showing the effects of the variables on the responses, (a) DT in sec and (b) *F* and DR in 25 minutes, where A is concentration of CCS (*X*_1_) and B is concentration of HRM (*X*_2_).

The resulting perturbation plots in Figure 3 help to competently study the effect of each independent factor at a certain point on a specific response, while the remaining factor maintained constant at a particular mentioned point. A steep or curve slope specifies that the response is sensitive to the definite variable. Figures 3(a)–3(c) show that the concentration of HRM (*X*_2_) had a maximum effect on DT, while concentration of CCS (*X*_1_) significantly affected *F* and DR. Rising level of *X*_2_ until the reference point results in decrease in DT (synergistic effect). Further increase of *X*_2_ after the reference point results in an increase in DT (antagonistic effect), whilst the elevated concentration of *X*_1_ had a synergistic effect on decrease in *F* and increases in DR.

### 4.5. Formation of 3D Response Surface Plots

**Figure 4:**
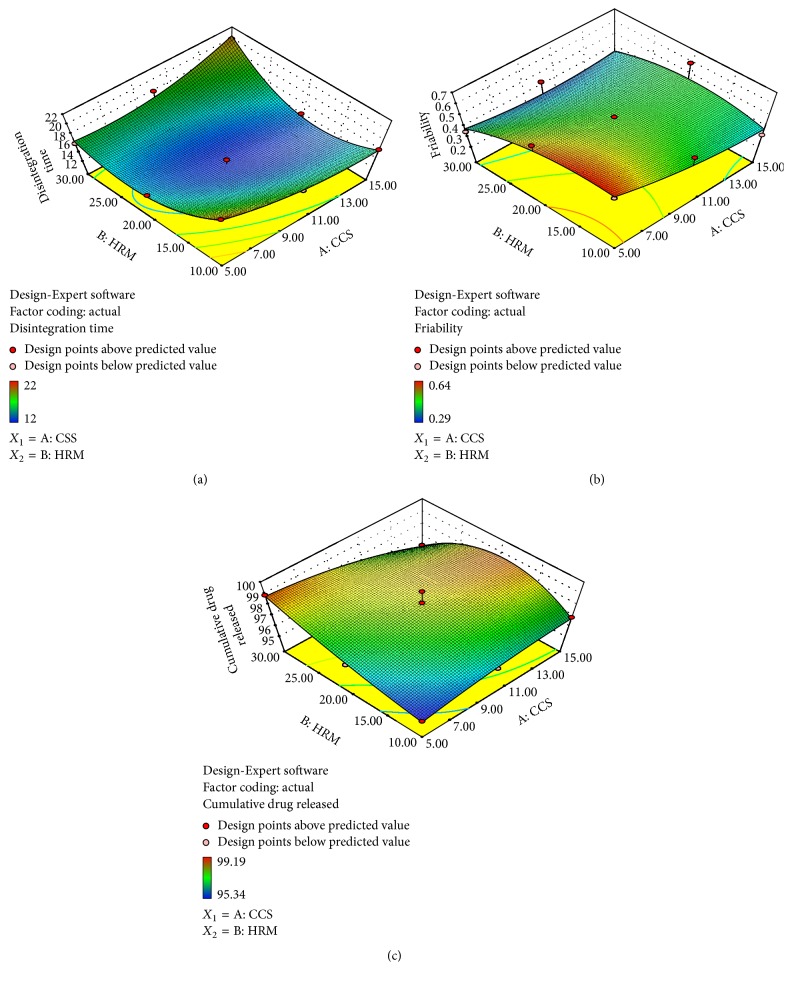
Response surface plot showing interaction of variable *X*_1_ (concentration of CCS) and variable *X*_2_ (concentration of HRM) influencing (a) disintegration time, (b) % friability, and (c) cumulative % drug release.

To envision the influence of independent factors on flux, three-dimensional (3D) plots in Figure 4 for (a) DT, (b) *F*, and (c) DR were formed based on the polynomial model. All of the observed response surfaces formed hillsides with large curvatures confirming that they were mostly influenced by the interaction effect of concentrations of HRM and CCS. From the response surface plots, it was concluded that the increase in concentration of both factors leads to significant decrease in DT, but at a certain point DT may increase due to the wicking problem.  Figure 4(a) exhibits that DT varies in a largely curved nonlinear descending order upon increasing HRM and decreasing CCS concentrations.  Figure 4(b) shows that *F* changes in a slightly curved linear descending manner when concentrations of HRM and CCS increased. However, it is illustrated that the DR (Figure 4(c)) alters in a largely curved nonlinear ascending model with increasing concentrations of HRM and CCS.

### 4.6. Optimization of the Model

From the above discussion, it fairly represents that the formulations of CTZ ODTs are very tough to forecast the overall output traits based on simple interpretation of significant factor deviation. Hence, the desirability function was employed to resolve the optimal default setting of the process parameters that will maximize the three responses. Optimization was performed to obtain the optimal values of *X*_1_ and *X*_2_ for achieving the desirability constraints in the range of disintegration time (12 to 14 seconds), % friability (0.29 to 0.5), and cumulative % drug release (99 to 101.32). The optimized amount of CCS and HRM was incorporated in Table 6.

### 4.7. Validation of RSM Results of CTZ ODTs Formulation

**Figure 5:**
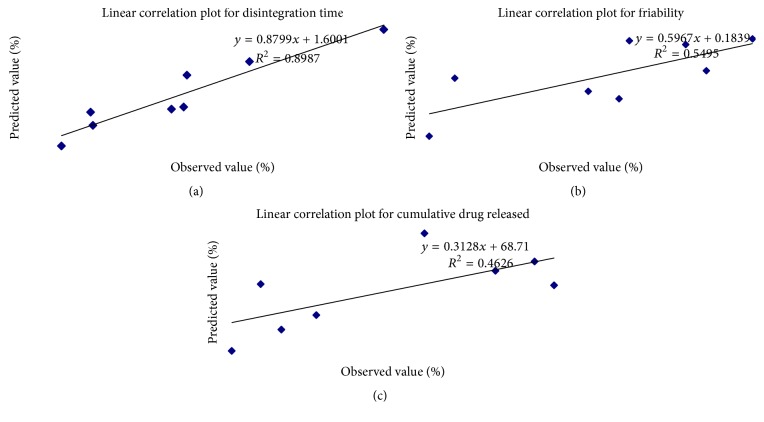
Linear correlation plots between observed and predicted values for (a) DT in sec, (b) percent of *F*, and (c) percent of cumulative drug release of cetirizine HCl oral disintegration tablet formulations.

Eight checkpoint solutions were selected on the principles of optimal formulation specified by thorough grid search to validate the selected experimental design and nonlinear polynomial equations. These checkpoint solutions were prepared and evaluated for the dependent response properties.  Table 7 lists the composition, experimental and predicted values of the three response variables, and their respective percentage errors. Moreover, the linear regression plots were constructed accurately between observed and predicted values to the individual response characteristics by MS-Excel, imposing the trend-line all the way through starting point to measure quantitatively as shown in Figures 5(a)–5(c). In the linear graph group of points are scattered above and below the 45° line showing better prediction and less error between experimental and predicted values. The formulation number *F∗*4 was optimized as the best formulation for CTZ ODTs in which the error was minimum for the dependent variables. The composition of optimized formulation *F∗*4 is given in Table 8. The powder flow properties and postcompression parameters are given in Tables 9 and 10, respectively.

## 5. Conclusion

In this experimental study, the synthetic and natural disintegrants were detected to have a reflective and collaborative influence upon the characteristics of ODTs of CTZ formulation. This system embraces the drug-delivery system that achieves fast and relatively quick release of the drug over agreeable period of time. The response variables of the formulation are optimized by RSM (CCD design), and the results observed indicated that this experimental design had been successfully applied to develop the combination of CCS and HRM to prepare ODTs with desirable rapid disintegration and drug release. Combination of synthetic and natural disintegrant using response surface methodology can be formulated in an ideal oral disintegrating tablet. Moreover, their mutual influences on studied parameters can be exploited and commercialized.

## Figures and Tables

**Table 1:** Physicochemical parameters of mucilage powder (number of experiments = 3).

Parameters	Conditions	
Angle of repose		26.37°
Bulk density		0.56 g/cm^3^
Tapped density		0.76 g/cm^3^
Average particle size		152 *µ*m
Compressibility ratio		26.31
Loss on drying		5%
Percentage yield		29%
Swelling ratio	In water	48
Solubility		Slowly soluble in water producing huge viscous solution
Total ash		20.35%
Acid insoluble ash		4.9%
Microbial load	Bacteria (CFUs/g)	6
	Fungi (CFUs/g)	3

**Table 2:** Composition of preliminary trial batch with individual disintegrant and their evaluation parameters.

Ingredients (mg)	*F*_A1_	*F*_A2_	*F*_A3_	*F*_A4_	*F*_A5_	*F*_A6_	*F*_A7_	*F*_A8_
CTZ	10	10	10	10	10	10	10	10
CCS	5	10	15	20	—	—	—	—
	(2.5%)	(5%)	(7.5%)	(10%)				
HRM	—	—	—	—	10	20	30	40
					(5%)	(10%)	(15%)	(20%)
Pearlitol SD 200	149	144	139	134	144	134	124	114
Sorbitol	20	20	20	20	20	20	20	20
Otherexcipients^*∗*^	12	12	12	12	12	12	12	12
Aspartame	4	4	4	4	4	4	4	4

Evaluation								
Parameters								

DT (sec)	31 ± 2	18 ± 2.2	25 ± 1.3	28 ± 2.3	39 ± 0.33	28 ± 0.12	36 ± 0.23	49 ± 0.32
*F* (%)	0.93	0.91	1.11	1.34	0.49	0.31	0.79	0.87
Wetting time (sec)	27	16	24	22	36	25	33	47
DR at 25 min	98.24	98.99	98.72	97.47	93.32	98.32	89.45	87.33

Net tablet weight: 200 mg; batch size: 50 CTZ ODTs.

*Note.* The amount of all the ingredients was calculated on the basis of net weight of one tablet (200 mg). For the preliminary trial batch four varied concentrations of CCS and HRM (ΔCCS: 2.5%–10%; ΔHRM: 5%–20%) were trialed for desired tablet properties. D-sorbitol (10%) and aspartame (2%) as sweetening agent were used to mask the inherent bitter taste of cetirizine hydrochloride in all the trial formulations.

^*∗*^Other excipients used were magnesium stearate, 2 mg (1%); aerosil, 2 mg (1%); talc, 2 mg (1%); and flavour, 6 mg (3%).

**Table 3:** Formulation trial carried out for oral disintegrating tablet formulation of CTZ ODTs with CCS/ HRM at different level as per experimental design.

Trial run	Coded factor levels						
		*X*_1_				*X*_2_	
*F* 1		−1				−1	
*F* 2		0				−1	
*F* 3		1				−1	
*F* 4		−1				0	
*F* 5		0				0	
*F* 6		1				0	
*F* 7		−1				1	
*F* 8		0				1	
*F* 9		1				1	
*F* 10		0				0	
*F* 11		0				0	
*F* 12		0				0	
*F* 13		0				0	

Translation coded values	−1			0			+1

*X*_1_: CCS (%)	2.5			5			7.5
*X*_2_: HRM (%)	5			10			15

Dependent variables							

*Y*_1_	Disintegration time (sec)						
*Y*_2_	Friability (%)						
*Y*_3_	Cumulative drug released at 25 minutes (%)						

**Table 4:** Response variables (*Y*_1_–*Y*_3_) obtained from trial formulations of CTZ ODTs.

Trial run	Croscarmellose sodium (mg) (*X*_1_)	*H. rosa-sinensis* mucilage (mg) (*X*_2_)	Disintegration time (sec) (*Y*_1_)	Friability (%) (*Y*_2_)	Cumulative drug released (%) (*Y*_3_)
*F*1	5	10	22	0.61 ± 0.16	95.34 ± 1.9
*F*2	10	10	17	0.53 ± 0.13	96.12 ± 2.56
*F*3	15	10	16	0.32 ± 0.07	96.87 ± 1.96
*F*4	5	20	15	0.64 ± 0.10	96.50 ± 1.77
*F*5	10	20	12	0.47 ± 0.12	99.19 ± 2.24
*F*6	15	20	14	0.59 ± 0.09	98.50 ± 1.35
*F*7	5	30	16	0.35 ± 0.17	98.88 ± 1.71
*F*8	10	30	18	0.43 ± 0.03	97.76 ± 2.55
*F*9	15	30	20	0.29 ± 0.11	96.22 ± 1.33
*F*10	10	20	13	0.41 ± 0.30	97.39 ± 1.95
*F*11	10	20	12	0.39 ± 0.21	97.98 ± 1.28
*F*12	10	20	12	0.46 ± 0.05	98.01 ± 1.25
*F*13	10	20	12	0.49 ± 0.25	98.2 ± 1.11

**Table 5:** ANOVA for response surface quadratic model for responses.

Term	DT		*F*		DR	
	*F* value	*p* value	*F* value	*p* value	*F* value	*p* value
Model	46.08	0.0003^*∗*^	1.25	0.0156^*∗*^	7.52	0.0205^*∗*^
*X*_1_-CCS	1.27	0.3106	0.12	0.7457	2.46	0.1777
*X*_2_-HRM	1.27	0.3106	0.47	0.5235	0.77	0.4191
*X*_1_ *X*_2_	63.60	0.0005^*∗*^	1.24	0.3156	8.45	0.0335^*∗*^
*X*_1_^2^	19.65	0.0068^*∗*^	0.55	0.4901	0.15	0.7184
*X*_2_^2^	153.39	<0.0001^*∗*^	2.05	0.2120	31.92	0.0024^*∗*^
*X*_1_^2^ *X*_2_	3.39	0.1249	0.063	0.8112	0.011	0.9192
*X*_1_ *X*_2_^2^	0.000	1.0000	0.49	0.5154	0.82	0.4075

^*∗*^ *P* < 0.05 gives an indication of the significance of an effect *α* = 0.05.

**Table 6:** Optimization of CTZ ODTs formulation by surface response method.

	Constraints		
Name	Goal	Lower limit	Upper limit
CCS	In range	−1	1
HRM	In range	−1	1
DT (sec)	In range	12	14
*F* (%)	In range	0.29	0.5
DR in 25 minutes	In range	99.0	101.32

**Table 7:** Composition of the checkpoint formulations and the predicted and experimental values of response variables.

Number	Croscarmellose sodium (mg)	*H. rosa-sinensis* mucilage (mg)	Response variables	Observed response	Predicted response	Percentage error	Avg
			Disintegration time	12.392	12.361	0.031	
*F∗*1	10.34	20.11	% friability	0.446	0.478	−0.032	2.231
			Cumulative % drug released	100.031	100.367	−0.336	

			Disintegration time	12.932	12.834	0.763	
*F∗*2	9.13	17.36	% friability	0.511	0.496	3.024	1.008
			Cumulative % drug released	100.64	99.986	0.654	

			Disintegration time	12.993	12.861	1.026	
*F∗*3	8.72	17.92	% friability	0.524	0.499	5.01	1.67
			Cumulative % drug released	99.261	99.995	−0.734	

			Disintegration time	13.010	13.271	−1.96	
*F∗*4	9.05	16.04	% friability	0.500	0.498	0.401	**0.133**
			Cumulative % drug released	99.523	99.768	−0.245	

			Disintegration time	13.323	13.445	−0.907	
*F∗*5	10.61	14.23	% friability	0.492	0.471	4.458	1.48
			Cumulative % drug released	99.358	99.661	−0.304	

			Disintegration time	12.539	12.627	−0.696	
*F∗*6	10.40	16.71	% friability	0.515	0.482	6.846	2.282
			Cumulative % drug released	100.364	100.092	0.272	

			Disintegration time	12.528	12.796	−2.094	
*F∗*7	12.14	15.49	% friability	0.498	0.467	6.638	0.212
			Cumulative % drug released	100.548	100.161	0.386	

			Disintegration time	13.995	13.857	0.995	
*F∗*8	11.50	13.00	% friability	0.461	0.447	3.131	1.284
			Cumulative % drug released	99.125	99.505	−0.381	

**Table 8:** Final optimized formulation (*F∗*4) of CTZ ODTs formulation.

Composition	Amount
CTZ	10 mg
Pearlitol SD 100	128.91 mg
CCS	9.05 mg
HRM	16.04 mg
Sorbitol	20 mg
Other excipients	12 mg
Aspartame	4 mg

^*∗*^Other excipients: magnesium stearate: 2 mg, aerosil: 2 mg, talc: 2 mg, flavour: 6 mg, total tablet weight: 200 mg, and batch size: 50 tablets.

**Table 9:** Powder flow properties of the optimized formulation (*F∗*4).

Properties	Optimized formulation (*F∗*4)
Angle of repose	26.46
Bulk density	0.487
Tapped density	0.581
Carr's Compressibility Index	16.71
Hausner's ratio	1.192

**Table 10:** Postcompression parameters of the optimized formulation (*F∗*4).

Parameters	Value
Wetting time (sec)^*∗∗*^	10
Water absorption ratio (%)^*∗∗*^	89.01 ± 0.05
Weight variation (%)^#^	0.71
Thickness (mm)^*∗*^	3.1 ± 0.28
Hardness	3.1 ± 0.44
Content uniformity (%)^#^	99.99 ± 0.35
Residual remaining on the screen ≠ 22^#^	No
Average weight^*∗∗*^	199.81
Taste/mouth feel^*∗*^	Palatable

^**∗**^Each value was an average of six determinations. ^**∗∗**^Each value was an average of three determinations. ^#^Results of one batch *n* = 20.

## References

[1] Xu J., Bovet L. L., Zhao K. (2008). Taste masking microspheres for orally disintegrating tablets. *International Journal of Pharmaceutics*.

[2] Seager H. (1998). Drug-delivery products and the Zydis fast-dissolving dosage form. *Journal of Pharmacy and Pharmacology*.

[3] Dobetti L. (2000). Fast melting tablets: developments and technologies. *Pharmaceutica. Technology Europe*.

[4] Hamlen S., MacGregor K. (2011). Patient compliance study: new data shows drug delivery has positive impact on patient compliance. *Drug Development & Delivery*.

[5] Guhmann M., Preis M., Gerber F., Pöllinger N., Breitkreutz J., Weitschies W. (2012). Development of oral taste masked diclofenac formulations using a taste sensing system. *International Journal of Pharmaceutics*.

[6] Abdelbary G., Prinderre P., Eouani C., Joachim J., Reynier J. P., Piccerelle P. (2004). The preparation of orally disintegrating tablets using a hydrophilic waxy binder. *International Journal of Pharmaceutics*.

[7] Singh S., Shah D. (2012). Development and characterization of mouth dissolving tablet of zolmitriptan. *Asian Pacific Journal of Tropical Disease*.

[8] Late S. G., Yu Y.-Y., Banga A. K. (2009). Effects of disintegration-promoting agent, lubricants and moisture treatment on optimized fast disintegrating tablets. *International Journal of Pharmaceutics*.

[9] Ghosh A., Chakraborty P. (2013). Formulation and mathematical optimization of controlled release calcium alginate micro pellets of frusemide. *BioMed Research International*.

[10] Montgomery D. C. (1996). Response surface methodology. *Design and Analysis of Experiments*.

[11] Singh B., Chakkal S. K., Ahuja N. (2006). Formulation and optimization of controlled release mucoadhesive tablets of atenolol using response surface methodology. *AAPS PharmSciTech*.

[12] Singh B., Kumar R., Ahuja N. (2005). Optimizing drug delivery systems using systematic “design of experiments.” Part I: fundamental aspects. *Critical Reviews in Therapeutic Drug Carrier Systems*.

[13] Singh B., Dahiya M., Saharan V., Ahuja N. (2005). Optimizing drug delivery systems using systematic ‘design of experiments.’ Part II: retrospect and prospects. *Critical Reviews in Therapeutic Drug Carrier Systems*.

[14] Sweetman S. C. (2007). *Martindale: The Complete Drug Reference*.

[15] Mishra R., Amin A. (2011). Formulation and characterization of rapidly dissolving films of cetirizine hydrochloride using pullulan as a film forming agent. *Indian Journal of Pharmaceutical Education and Research*.

[16] Prabakaran L., Murthy V. S. N., Karpakavalli M. (2011). Extraction and characterization of Hybiscus Rosa-Sinensis leaves mucilage for Pharmaceutical applications. *RGUHS Journal of Pharmaceutical Sciences*.

[17] Ahad H. A., Kumar C. S., Yesupadam P., Rani P. S., Sekhar A. C., Sivaramakrishna G. V. (2010). Fabrication and characterization of diclofenac sodium hibiscus rosa-sinensis leaves mucilage sustained release matrix tablets. *Der Pharmacia Lettre*.

[18] Schiermeier S., Schmidt P. C. (2002). Fast dispersible ibuprofen tablets. *European Journal of Pharmaceutical Sciences*.

[19] Basak S. C., Reddy B. M. J., Mani K. P. L. (2006). Formulation and release behaviour of sustained release ambroxol hydrochloride HPMC matrix tablet. *Indian Journal of Pharmaceutical Sciences*.

[20] Chopra S., Patil G. V., Motwani S. K. (2007). Release modulating hydrophilic matrix systems of losartan potassium: optimization of formulation using statistical experimental design. *European Journal of Pharmaceutics and Biopharmaceutics*.

[21] Radhika P. R., Pal T. K., Sivakumar T. (2009). Optimization of glipizide sustained release matrix tablet formulation by central composite design- response surface methodology. *Journal of Pharmacy Research*.

[22] Sahoo B. K., Chakraborty U., Mukherjee J., Pal T. K. (2010). Optimization and validation of modulated release formulation of ranitidine HCl by response surface methodology. *Journal of Biomedical Sciences and Research*.

[23] Bi Y. X., Sunada H., Yonezawa Y., Danjo K. (1999). Evaluation of rapidly disintegrating tablets prepared by a direct compression method. *Drug Development and Industrial Pharmacy*.

[24] Jones R. J., Rajabi-Siahboomi A., Levina M., Perrie Y., Mohammed A. R. (2011). The influence of formulation and manufacturing process parameters on the characteristics of lyophilized orally disintegrating tablets. *Pharmaceutics*.

[25] Douroumis D. D., Gryczke A., Schminke S. (2011). Development and evaluation of cetirizine HCl taste-masked oral disintegrating tablets. *AAPS PharmSciTech*.

[26] Chang J.-S., Huang Y.-B., Hou S.-S., Wang R.-J., Wu P.-C., Tsai Y.-H. (2007). Formulation optimization of meloxicam sodium gel using response surface methodology. *International Journal of Pharmaceutics*.

[27] Ahuja M., Yadav M., Kumar S. (2010). Application of response surface methodology to formulation of ionotropically gelled gum cordia/gellan beads. *Carbohydrate Polymers*.

[28] Pabari R. M., Ramtoola Z. (2012). Application of face centred central composite design to optimise compression force and tablet diameter for the formulation of mechanically strong and fast disintegrating orodispersible tablets. *International Journal of Pharmaceutics*.

[29] Sunada H., Bi Y. (2002). Preparation, evaluation and optimization of rapidly disintegrating tablets. *Powder Technology*.

[30] Gan H. E., Karim R., Muhammad S. K. S., Bakar J. A., Hashim D. M., Rahman R. A. (2007). Optimization of the basic formulation of a traditional baked cassava cake using response surface methodology. *LWT—Food Science and Technology*.

[31] Furlanetto S., Cirri M., Maestrelli F., Corti G., Mura P. (2006). Study of formulation variables influencing the drug release rate from matrix tablets by experimental design. *European Journal of Pharmaceutics and Biopharmaceutics*.

